# Evidence-Based High-Loading Tendon Exercise for 12 Weeks Leads to Increased Tendon Stiffness and Cross-Sectional Area in Achilles Tendinopathy: A Controlled Clinical Trial

**DOI:** 10.1186/s40798-022-00545-5

**Published:** 2022-12-20

**Authors:** Goran Radovanović, Sebastian Bohm, Kim Kristin Peper, Adamantios Arampatzis, Kirsten Legerlotz

**Affiliations:** 1 Institute of Sports Sciences, Movement Biomechanics, Humboldt-Universität zu Berlin, Philippstr. 13, 10115 Berlin, Germany; 2 Department Performance, Neuroscience, Therapy and Health, Medical School Hamburg, Faculty of Health Sciences, University of Applied Sciences and Medical University, 20457 Hamburg, Germany; 3 Institute of Interdisciplinary Exercise Science and Sports Medicine, Medical School Hamburg, University of Applied Sciences and Medical University, 20457 Hamburg, Germany; 4 Department of Training and Movement Sciences, Humboldt-Universität zu Berlin, 10099 Berlin, Germany; 5 Munich Institute of Robotics and Machine Intelligence, Technische Universität München, 80992 Munich, Germany

**Keywords:** Achilles tendinopathy, Controlled clinical trial, Tendon adaptation, High-loading exercise, Musculoskeletal rehabilitation, Tendon rehabilitation, Training therapy, Eccentric training, Physiotherapy

## Abstract

**Background:**

Assuming that the mechanisms inducing adaptation in healthy tendons yield similar responses in tendinopathic tendons, we hypothesized that a high-loading exercise protocol that increases tendon stiffness and cross-sectional area in male healthy Achilles tendons may also induce comparable beneficial adaptations in male tendinopathic Achilles tendons in addition to improving pain and function.

**Objectives:**

We investigated the effectiveness of high-loading exercise in Achilles tendinopathy in terms of inducing mechanical (tendon stiffness, maximum strain), material (Young’s modulus), morphological (tendon cross-sectional area (CSA)), maximum voluntary isometric plantar flexor strength (MVC) as well as clinical adaptations (Victorian Institute of Sports Assessment—Achilles (VISA-A) score and pain (numerical rating scale (NRS))) as the primary outcomes. As secondary outcomes, drop (DJ) and counter-movement jump (CMJ) height and intratendinous vascularity were assessed.

**Methods:**

We conducted a controlled clinical trial with a 3-month intervention phase. Eligibility criteria were assessed by researchers and medical doctors. Inclusion criteria were male sex, aged between 20 and 55 years, chronic Achilles tendinopathy confirmed by a medical doctor via ultrasound-assisted assessment, and a severity level of less than 80 points on the VISA-A score. Thirty-nine patients were assigned by sequential allocation to one of three parallel arms: a high-loading intervention (training at ~ 90% of the MVC) (*n* = 15), eccentric exercise (according to the Alfredson protocol) as the standard therapy (*n* = 15) and passive therapy (*n* = 14). Parameters were assessed pre- and-post-intervention. Data analysis was blinded.

**Results:**

Primary outcomes: Plantar flexor MVC, tendon stiffness, mean CSA and maximum tendon strain improved only in the high-loading intervention group by 7.2 ± 9.9% (*p* = 0.045), 20.1 ± 20.5% (*p* = 0.049), 8.98 ± 5.8% (*p* < 0.001) and −12.4 ± 10.3% (*p* = 0.001), respectively. Stiffness decreased in the passive therapy group (−7.7 ± 21.2%; *p* = 0.042). There was no change in Young’s modulus in either group (*p* > 0.05). The VISA-A score increased in all groups on average by 19.8 ± 15.3 points (*p* < 0.001), while pain (NRS) dropped by −0.55 ± 0.9 points (*p* < 0.001). Secondary outcomes: CMJ height decreased for all groups (−0.63 ± 4.07 cm; *p* = 0.005). There was no change in DJ height and vascularity (*p* > 0.05) in either group.

**Conclusion:**

Despite an overall clinical improvement, it was exclusively the high-loading intervention that induced significant mechanical and morphological adaptations of the plantar flexor muscle–tendon unit. This might contribute to protecting the tendon from strain-induced injury. Thus, we recommend the high-loading intervention as an effective (alternative) therapeutic protocol in Achilles tendinopathy rehabilitation management in males.

*Clinical Trials Registration Number*: NCT02732782.

## Key Points

- In a controlled clinical trial, high-loading intervention in Achilles tendinopathy in males induced superior adaptations in tendon stiffness, maximum tendon strain and cross-sectional area as well as similar clinical improvements when compared to standard eccentric exercise or passive therapy.
- High-loading exercise-induced adaptations may further lead to prolonged benefits as improved mechanical and morphological tendon properties might protect the tendon from strain-induced microdamage and pain.
- We recommend the high-loading intervention as an effective (alternative) therapeutic protocol in Achilles tendinopathy rehabilitation management.

## Introduction

Achilles tendinopathy, characterized by swelling, load-induced pain and loss of function [1], is the most prevalent occurring tendinopathy of the lower extremity [2]. While a variety of different treatment approaches are available [3, 4], many of those exclusively focus on symptoms such as pain, yet do not address the underlying physiological causes.

The exact etiology remains unclear [5, 6], and it is likely to be multifactorial, including metabolic dysfunction (diabetes) [7] and hypercholesterolemia [8], or overstimulated inflammatory processes [9]. Despite this, a predominant cause from a mechanobiological perspective seems to be repetitive mechanical tissue overuse [6, 10]. Particularly, repetitive strain beyond physiological limits is linked to tendon injury [11–13]. As a consequence, tendon overuse may result in structural damage leading to a degenerated and weakened tendon [14] with reduced capacity to resist deformation when force is applied (i.e., decrease in stiffness).

A temporary insufficient tissue capacity that increases the risk for tendon injury may apply particularly to athletes, where non-uniform maturation and adaptation of muscle and tendon tissue [15, 16] can add to the observed considerable alterations in tendon stiffness across a season [17]. Indeed, the prevalence of tendinopathy in specific sports is high, with up to 50% in professional endurance runners [18–20] and handball [21], basketball [22] and volleyball [23] players. However, repetitive mechanical loading does not necessarily lead to tendon injury [24], as loading is also needed to maintain tissue function and homeostasis [11] and may lead to tendon tissue adaptation (i.e., increase in stiffness). As an example, tendon stiffness is increased in sprinters compared to endurance athletes or controls [25], while elite jumpers show higher stiffness in their take-off leg compared to their swing leg [17]. Indeed, there seems to be a thin line between the benefits and detriments of mechanical loading in terms of strain to the tendon [10].

The specific characteristics of the mechanical stimulus play a key role in the context of adaptation: repetitive high tendon strain induced by highly intensive muscle contractions (e.g., at 90% of maximum voluntary isometric contraction (MVC)) and a time under tension of at least three seconds are proposed to be effective for tissue adaptive responses, leading to stiffness increases and tissue growth in healthy tendons [26–28]. In contrast, lower tendon strains induced by medium intensive muscle contractions (e.g., 55% of MVC [26, 27]) or treadmill walking and running [29] as well as short time under tension as occurring in plyometric jumps [28] did not lead to similar adaptive responses. Thus, to counteract phases of enhanced injury risk, a specific tendon training based on the most effective mechanical stimulus should improve the structural integrity of the tendon and thereby reducing injury prevalence. Indeed, high-loading exercise decreased the prevalence of tendon pain in adolescent handball and basketball players [30, 31]. Taking the aforementioned into consideration, it seems reasonable to assume that tendinopathy patients in which the tendons tissue integrity has deteriorated may also benefit from high-loading interventions on a structural and clinical level.

While the therapeutical use of mechanical stimuli [32] has been standard in tendinopathy treatment for many years, effectively improving pain and function in many patients, exercise therapies such as eccentric calf muscle training, known as the Alfredson protocol, [33] do not lead to pronounced tendon stiffness adaptations [34–36]. The effects of eccentric exercise on tendon CSA remain ambiguous as one study showed an increase in tendon CSA [36] whereas another study did not [37].

Yet, a recent trial in patellar tendinopathy that compared eccentric exercise to progressive-tendon-loading exercise (PTLE) demonstrated promising evidence for the superior clinical outcome (i.e., VISA-P score) with PTLE treatment [38]. Despite this, from a biomechanical viewpoint, the effectiveness of an exercise treatment protocol is linked to its capacity in inducing structural tendon adaption. Indeed, 12 weeks of heavy-slow-resistance training in patellar tendinopathy induced changes in the extracellular matrix composition, indicating an increased collagen synthesis and turnover but without altering the mechanical (i.e., stiffness), material (i.e., Young’s modulus) and morphological (i.e., CSA) patellar tendon properties [36]. The latter might be supported by a recent trial in which either low-loading or high-loading exercise did not lead to improvements in mechanical, material, or morphological tendon properties in patellar tendinopathy [39].

In Achilles tendinopathy, 12 weeks of heavy load resistance training did not result in superior effects in VISA-A score, pain, tendon thickness and vascularization compared to eccentric exercise [40]. However, comprehensive assessments of the Achilles tendon mechanical, material, or morphological properties were not performed in that study and those data are still missing.

Thus, we hypothesize that a mechanical loading protocol that induces adaptation in healthy Achilles tendons [28] yields similar responses in tendinopathic Achilles tendons, meaning that a high-loading exercise protocol that increases tendon stiffness, CSA [28] and DJ performance [41] in a healthy population should also induce beneficial structural adaptations in tendinopathic Achilles tendons in addition to improving pain and function, counteracting the pathological tissue deterioration. In addition, the high-loading protocol is highly time-saving as it requires approximately 20% of the time when compared to the Alfredson protocol (Table 1).

**Table 1:** Baseline characteristics of the three groups and intervention characteristics of the two exercise protocols

Baseline characteristics	Passive therapy (*n* = 14)	Alfredson (*n* = 15)	High-load (*n* = 15)
Age [years]	42 ± 11 (26–55)	40 ± 8 (24–52)	39 ± 9 (25–52)
Body height [cm]	182 ± 6 (173–195)	179 ± 5 (170–186)	184 ± 6 (177–198)
Body mass [kg]	80 ± 12 (68–110)	76 ± 7 (65–88)	85 ± 11 (70–110)
Body mass index [kg/m^2^]	24 ± 4 (20–37)	24 ± 2 (21–28)	25 ± 3 (22–32)
Activity level [hours/week]^A^	6 ± 4 (0–14)	6 ± 4 (0–12)	7 ± 6 (1–23)
Laterality (left/right)	3/11	2/12^B^	3/11^B^
Symptomatic AT (left/right)	10/4	7/8	9/6
Symptom localization (Ins./Mid-portion)	5/9	7/8	7/8
Symptom duration [months]	17 ± 20 (4–60)	45 ± 68 (3–264)	25 ± 33 (3–96)
VISA-A PRE [points]	59 ± 15 (19–78)	53 ± 14 (29–73)	57 ± 11 (35–73)
*Intervention characteristics*			
Loading time per session [min]	–	4.5	1
Loading time per week [min]	–	63	4
Time per session [min]^C,D^	–	11.75	5.75
Time per week [min]^C,D^	–	164.5	23

Values of baseline characteristics are presented as mean ± SD (standard deviation) and range in parentheses

Ins., Insertional; AT, Achilles tendon; VISA-A, Victorian Institute of Sport Assessment questionnaire score for Achilles tendinopathy

^A^Average of the 2 weeks before baseline

^B^*n* = 1 missing

^C^Rest periods added

^D^Without warm-up

We conducted a controlled clinical trial with Achilles tendinopathy patients hypothesizing that high-loading tendon exercise would provide superior therapeutic effects in terms of beneficial mechanical, material, and morphological adaptations of the tendon, plantar flexor strength and clinical parameters (i.e., primary outcomes) as well as functional parameters (i.e., vertical jump height) and tendon vascularity (i.e., secondary outcomes) when compared to common standard exercise therapy (Alfredson’s eccentric exercise protocol) or passive therapy (i.e., no mechanical loading).

## Materials and Methods

### Design

**Fig. 1:**
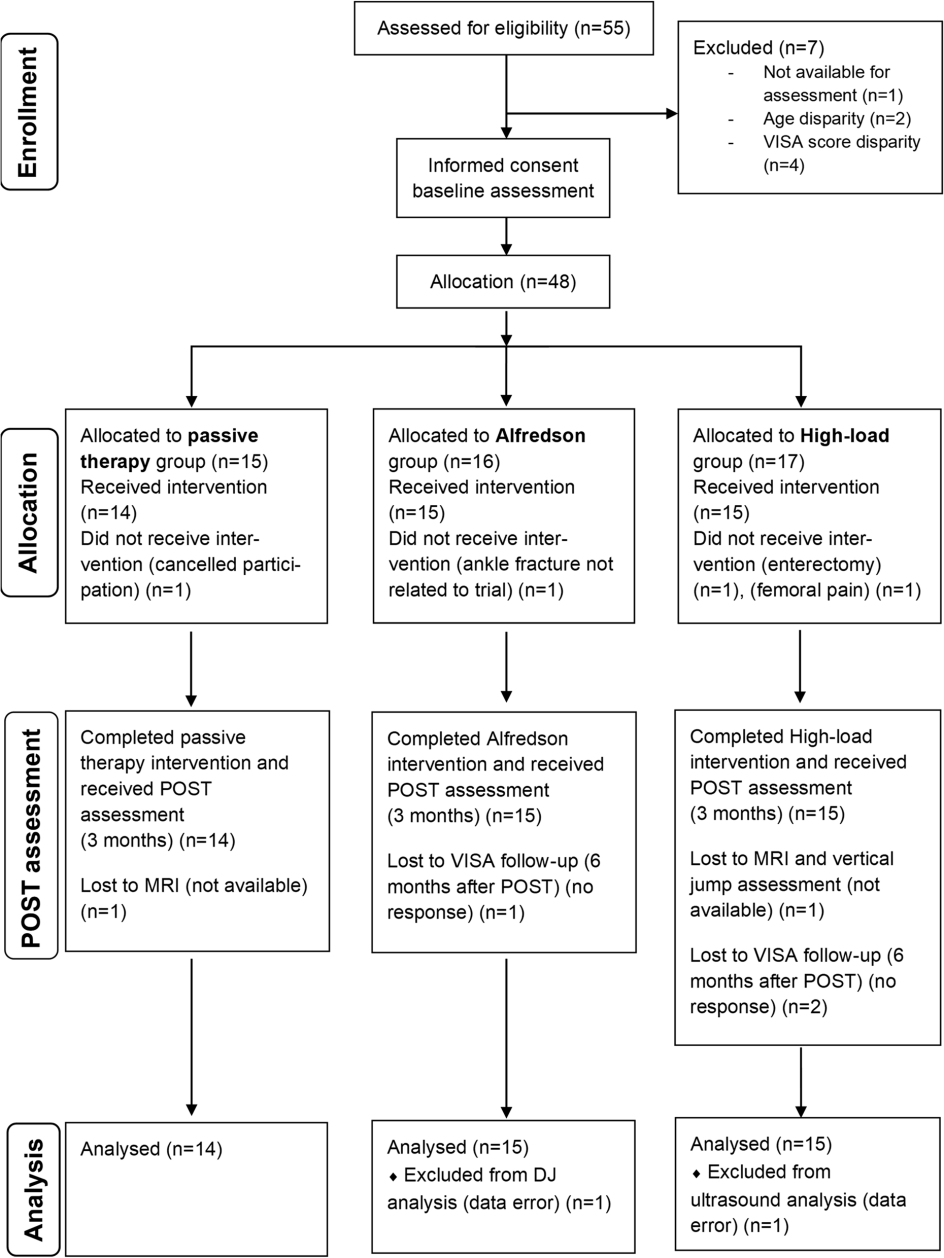
Participant flowchart according to the CONSORT (Consolidated Standards of Reporting Trials) guidelines [42]. At baseline and after completion of the 12-week intervention phase, we assessed the mechanical, material, morphological, functional, and clinical properties of the muscle–tendon complex. VISA = Victorian Institute of Sport Assessment questionnaire; MRI = Magnetic Resonance Imaging; DJ = drop jump

**Fig. 2:**
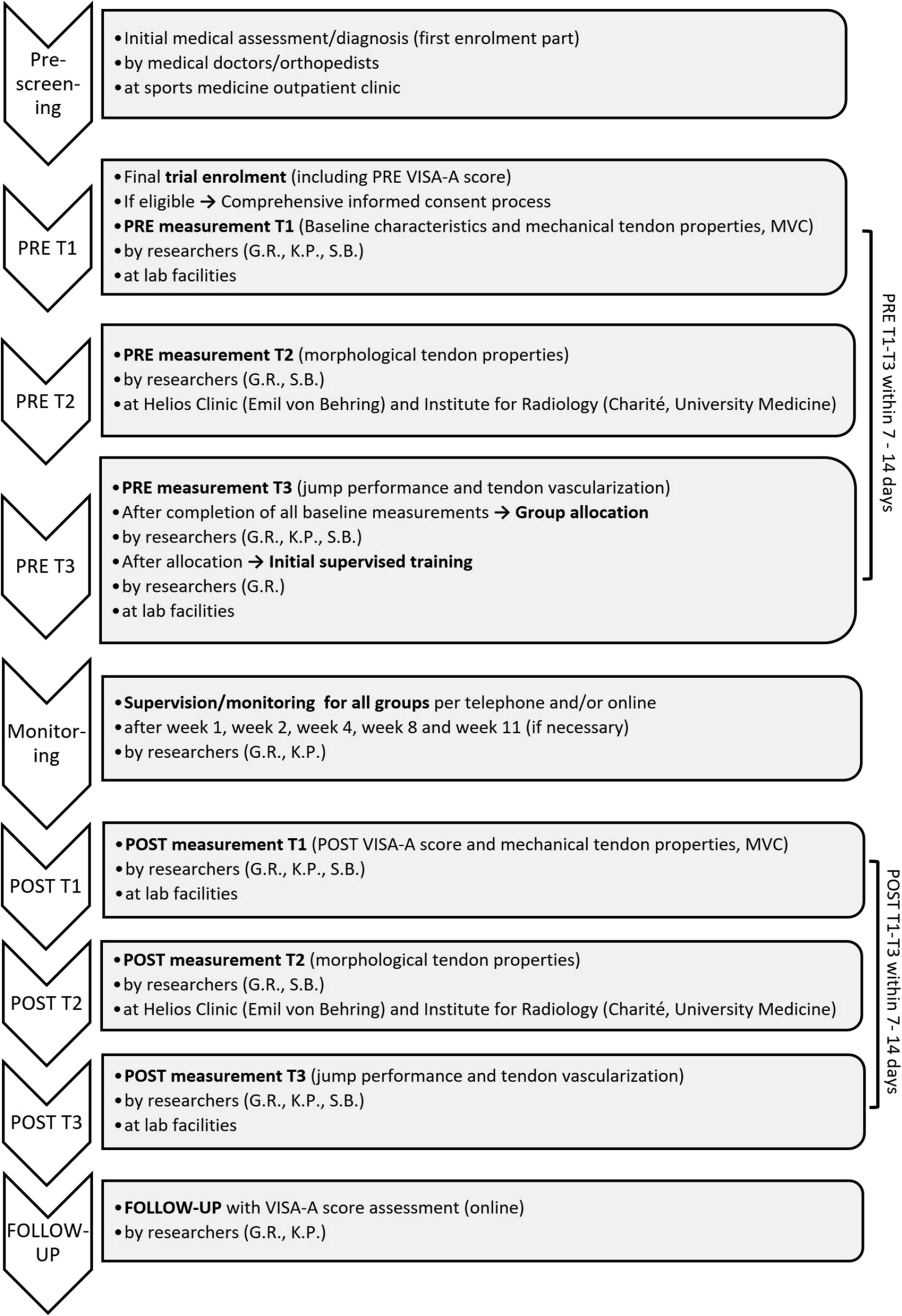
Data collection timeline flowchart. PRE = before the intervention phase, *T* = timepoint, VISA-A = Victorian Institute of Sports Assessment—Achilles, POST = after completion of 12 weeks of intervention, FOLLOW-UP = 6 months after completion of the intervention phase

The study design was a single-assessor-blinded (i.e., data analysis) controlled clinical parallel-arm trial with an intervention phase of 12 weeks, registered under *ClinicalTrials.gov* (NCT02732782). The study was conducted according to the CONSORT (Consolidated Standards of Reporting Trials) guidelines [42] (Fig. 1). We recruited patients by physician referral or advertisement (from 05/16 to 12/17). The pre-screening (i.e., initial medical assessment and diagnosis) by medical doctors took place at the sports medicine outpatient clinic of the Charité University Hospital, Berlin. Except for magnetic resonance imaging (MRI), all assessments and evaluations at baseline (PRE) and after completion of the intervention phase (POST) were conducted within the lab facilities of the Department of Training and Movement Sciences at the Humboldt-Universität zu Berlin (G.R., S.B. and K.P.) (Fig. 2). The assessment 6 months after POST (FOLLOW-UP) was performed online.

Eligibility criteria were assessed by researchers (G.R., S.B. and K.P.) within the laboratory facilities and orthopedic physicians from the sports medicine outpatient clinic (Charité University Hospital, Berlin) (Fig. 2). Inclusion criteria were male, aged between 20 and 55 years, and chronic condition (> 3 months) of Achilles tendinopathy. Pathology had to be confirmed via ultrasound (at least discrete hypo-echogenic areas within the tendon) and clinical assessment by a medical doctor. The threshold level of severity was defined by the Victorian Institute of Sport Assessment for Achilles tendinopathy (VISA-A) score of less than 80 points [43]. Exclusion criteria were corticosteroid infiltration of the tendon or any intake of antibiotics (such as Fluoroquinolone, Levofloxacin, Ciprofloxacin) [44] within the past 12 months, any leg surgery, tendon rupture or signs of partial rupture, any systemic inflammatory condition (e.g., rheumatoid arthritis, diabetes) and any spondyloarthropathies (e.g., spondylitis ankylosans). In cases of bilateral symptoms, the leg with a lower clinical (i.e., VISA-A) score and higher pain level was chosen. As there have not been any studies assessing tendon stiffness and Young’s modulus, particularly in Achilles tendinopathy using our high-loading tendon protocol, we relied on prior statistical power analysis in two studies with healthy subjects [26, 27]. This power analysis calculated a sample size of at least *n* = 12 per group to achieve high statistical power (*α* = 0.05, power = 0.95, correlation = 0, effect size: stiffness 1.6, Young’s modulus 1.2) [28]. Anticipating a dropout of approximately 20%, we decided to aim for 15 patients per group.

Fifty-five males registered interest and seven of them were excluded during the enrollment process (Fig. 1). Forty-eight participants met the eligibility criteria. They were enrolled in the study and after full completion of the PRE measurements (G.R., S.B. and K.P.) assigned by G.R. to one of the three treatment groups by an ABC pattern based on the order of date of pre-screening: The passive therapy group obtaining passive therapy sessions (i.e., no lower-limb mechanical loading), the standard exercise treatment (i.e., Alfredson group performing home-based eccentric exercise) and the High-load group conducting home-based high-loading tendon exercise. Four participants dropped out during the intervention phase (dropout rate of 8.33%). The remaining 44 participants were allocated as follows: Passive therapy group (*n* = 14), Alfredson group (*n* = 15) and High-load group (*n* = 15) (Table 1).

#### Allocation and Blinding

The allocation sequence list was generated, possessed, and hidden only by the study organizing researcher (G.R.). Except for him (G.R.), the allocation sequence was concealed for every other person involved in the enrollment, allocation, and baseline assessment process (i.e., medical doctors, researchers, assessors, data analysts and patients). Only after having finished baseline assessments PRE T1–T3, the respective assessor was informed by the researcher (G.R.) about the forthcoming allocation (Fig. 2). The chronological order of the patients at PRE-SCREENING did not correspond to the chronological order of PRE T1-3. Thus, allocation-to-group was not predictable by chronological order at any timepoint from PRE T1 -3. Allocation to groups, PRE measurements, supervision and POST measurements were carried out with strict adherence to standardized assessment procedures to all groups equally without disclosure of our study hypotheses. All PRE and POST assessed and reported data were gathered anonymously and without allocation information, and thus all image processing and data analysis was blinded.

### Intervention

All patients equally received the option for 12 therapeutic appointments (i.e., manual therapy, tissue and/or joint mobilization) as a prescription according to the national medical guidelines with free choice of location. The prescription stated the clinical trial involvement with recommendations for the physiotherapists to apply passive treatments and to refrain from active plantar flexor strength training, especially excluding eccentric exercises during the intervention phase. These recommendations were additionally forwarded to the participants by a *letter to the physio*. Patients were thoroughly informed about the intervention modalities by an experienced physiotherapist (G.R.) in addition to receiving a hand-out with clear and detailed instructions of the intervention protocol. During the intervention phase, patients were monitored and supervised on week 1, week 2, week 4, week 8 and week 11 via telephone call and/or email to ensure compliance (G.R. and K.P.). Moreover, all patients received a training diary for daily recording intervention training frequency, training load and its progression, pain on an NRS scale from 0 to 10 as a mean per day, use of medication, physiotherapy frequency and treatment content, further exercise activities (in km or in min) and additional comments. The activity was assessed in hours per week and based on verbal information (i.e., at PRE T1) as the baseline value (average of the two weeks before PRE T1) and based on the training diary (for PRE and POST). In case of ambiguity (i.e., distance report instead of time within the diary), we converted 10 km of running and 30 km of biking to 1-h activity. Activities like slow strolling, swimming, hiking, skiing, and slow commuting by bike were excluded from activity quantification. Compliance was defined as the percentage of the prescribed intervention.

All patients were allowed to continue with their training habits unless it induced Achilles tendon pain with a level of > 3/10 (NRS scale) up to 24 h later. No additional strength training of the plantar flexors and no implementation of any new sort of lower body training was permitted.

*Passive therapy group*: Patients were asked to adhere to a maximum of 12 passive therapeutic and manual treatment sessions, while refraining from any explicit plantar flexor strength training or Alfredson eccentric exercise protocol for the time of intervention. Thus, full adherence was defined as having had 12 appointments.

*Alfredson group*: According to the commonly known and frequently published protocol [33], patients in the Alfredson group performed eccentric exercises in an upright standing position with only the forefoot of the injured leg on the edge of a stair lowering the heel with an eccentric phase of three seconds [45]. We ensured eccentric-only contractions of the plantar flexors by using the healthy leg to return to the start position. Moreover, the use of a full ankle angle of motion in the eccentric phase was encouraged. One session was defined as three sets of 15 repetitions with extended knees followed by another three sets of 15 repetitions with bended knees and 1-min rest in between sets. According to Alfredson, our protocol consisted of two sessions per day every day with no warm-up. The optional level of load progression was defined by a 5 kg additional load per week.

*High-load group*: Patients in the High-load group obtained a feedback-fitted sling (displaying the applied force due to an integrated strain gauge) for home-based application [41] of the high-loading protocol reported by Arampatzis et al. [26], which provides an efficient stimulus for tendon adaptation [27, 28, 46, 47]. Patients were instructed to sit on the floor with extended knees. The forefoot (with shoes) was placed in the foot plate. The ratchet was individually set and fixed as tightly as possible, to allow for maximal isometric plantar flexor contractions at a standardized ankle angle position (90°). For warm-up, the patients performed three sets of five isometric submaximal plantar flexor contractions with 3 s under tension followed by three sec of rest each and with a rest of 1-min in between sets. After the warm-up in the first supervised session, five isometric MVCs of the plantar flexors were executed. The individual training load was then calculated based on 90% of the mean of the five MVCs. After 10 min of rest, the first high-loading intervention exercise was conducted under supervision with five sets of four repetitions of 90% isometric MVC plantar flexor contractions with 3 s under tension followed by 3-s rest between repetitions and 1-min rest between sets. This training session was repeated four times per week for 12 weeks. The level of load progression was defined by ~ 5% of the individual training load per week.

*Alfredson group and High-load group*: Only the injured leg was trained. The following instructions were given referring to pain and load progression: No progression of training load within the first two weeks. The load can only be progressed once a week. Adapted by the pain-monitoring model from Silbernagel and Crossley [48], load progression was allowed when the pain level was < 6/10 (NRS scale) [40, 49] and individual rating of perceived exertion was < 3/10 (NRS scale) [48]. Load reduction was recommended when either the pain level was > 5/10 (NRS scale) [40, 49] or the individual rating of perceived exertion was > 5/10 (NRS scale) [48] and should be maintained for a whole week. In case of limited (impossible) load reduction, the repetition number and/or frequency should be reduced. The first session was supervised.

### Primary Outcomes

Primary outcomes were defined as the mechanical, material, and morphological Achilles tendon properties including plantar flexor muscle strength as well as the VISA-A score and pain.

#### Mechanical and Material Properties

Tendon mechanical and material properties (i.e., stiffness, CSA, Young’s modulus) were analyzed using dynamometry, electromyography (EMG), motion capture, ultrasonography and MRI.

For tendon stiffness assessment, patients were seated on a dynamometer (Biodex System 3, Biodex Medical Systems Inc., USA) with a fixed ankle angle in a neutral position (foot sole 90° perpendicular to the tibia), extended knee, hip angle of ~ 110° and the pelvis fixed with a rigid belt. After a standardized warm-up of up to ten moderate to submaximal voluntary isometric plantar flexor contractions and 1–3 MVCs [50], the patients conducted five ramped MVCs in order to achieve high reliability on tendon stiffness measurement [51] with a duration of five seconds each and 2-min rest between repetitions followed by 2–3 isometric plantar flexor MVCs with 2-min rest between repetitions. During all MVCs, standardized verbal encouragement during each attempt was given.

Stiffness was calculated based on the tendon force to tendon elongation ratio. To calculate Achilles tendon force, the plantar flexion moment was divided by the tendon lever arm. We measured the Achilles tendon lever arm with the tendon excursion method [52] by relating the displacement of the m. gastrocnemius medialis myotendinous junction (MTJ) assessed by B-mode ultrasound (7.5 MHz, My Lab60, Esaote, Genova, Italy) to the corresponding angular ankle joint excursion [53]. Changes in lever arm length during the contraction when compared to resting state were considered by including a corrective factor in our calculation [54].

The plantar flexor moment was calculated using an inverse dynamic approach taking the misalignment of the ankle joint axis to the dynamometer axis into consideration [55]. The inverse dynamic calculation was based on kinematic data from an infrared motion capture system (Vicon Nexus, version 1.7.1, Vicon Motion Systems, UK) integrating seven cameras operating at 250 Hz. The contribution of the antagonistic muscle to the plantar flexor moment was considered by determining the m. tibialis anterior activity during plantar flexor MVC with one pair of bipolar surface EMG electrodes (Myon m320RX, Myon AG, Switzerland, 1000 Hz) [56]. The antagonistic moment was estimated based on the relationship of the m. tibialis anterior EMG activity and the exerted moments during two submaximal isometric m. tibialis anterior contractions with slightly lower and higher activity than the m. tibialis anterior ramp contraction activity [56].

Achilles tendon elongation was assessed by placing a B-mode ultrasound probe within a custom-built foam cast that was fixed on the lower leg recording the MTJ displacement during the ramped MVCs. MTJ displacement was traced manually frame-by-frame within a custom-written MATLAB script (The MathWorks, version 2012, USA). To consider the effects of ankle joint motion on tendon elongation measurements, the passive displacement of the MTJ in relation to the ankle angle [57] was determined with five trials of slow passive (no muscle contraction) ankle joint motion over the full range of motion. Force and elongation data from five measurements each were averaged.

We calculated Achilles tendon stiffness as the slope of a linear regression of tendon force to tendon elongation between 50 and 100% of the maximum tendon force. Achilles tendon rest length was measured at 20° plantar flexion with extended knee [58] from the proximal posterior part of the tuber calcanei to the MTJ. We calculated the Young’s modulus of the Achilles tendon by multiplying tendon stiffness with the quotient of tendon rest length and tendon CSA. The stress of the Achilles tendon was determined as the ratio of tendon force and averaged Achilles tendon CSA (see chapter 2.3.2). Maximum tendon strain was calculated as the ratio of maximum tendon elongation (obtained during the ramp MVCs) to rest length.

#### Morphological Properties

We assessed the CSA of the free Achilles tendon either with a 0.25 T magnetic resonance imaging (MRI) scanner (G-Scan, Esaote, Italy) [3D hybrid contrast enhancement (HYCE) sequence, repetition time (TR) 10 ms, echo time (TE) 5 ms, flip angle 80°, slice thickness 3 mm, space between slices 0.4 mm] at Oscar-Helene-Heim Foundation, Helios clinic Emil von Behring, Berlin, Germany, or a 1.5 T MRI scanner (Siemens Avanto, Siemens, Germany) at the Institute for radiology, Charité – University Medicine, Berlin) [T1- weighted sequence, TR 460 ms, TE 20 ms, slice thickness 2 mm, space between slices 0.4 mm] using transversal and sagittal Achilles tendon scans. Every PRE-POST pair was analyzed with the same scanner. During MRI measurement, the standardized patient position was the supine position with extended hips and knees and the ankle joint fixed at 90°. The transversal scans were positioned perpendicular to the direction of the tibia and manually and assessor-blinded segmented using the software OsiriX (Pixmeo SARL, version 2.5.1, Switzerland) [59]. The sagittal scans were used to precisely determine the proximal (i.e., m. soleus–Achilles tendon junction) and distal (calcaneus bone insertion) end of the free Achilles tendon and its length. The length of the free Achilles tendon was calculated as a curved line through every digitalized transversal scan using a Delaunay triangulation [28]. Tendon cross-sectional area was determined in 10% increments across the whole free tendon length. Average Achilles tendon CSA was calculated as a mean of all assessed CSAs of the free Achilles tendon.

#### Clinical Outcomes

As a patient-reported outcome measurement (PROM), we used the reliable and valid VISA-A score [60] to determine clinical severity. The VISA-A score was evaluated PRE (in-person), POST (in-person), and FOLLOW-UP (online). According to Stevens and Tan [61], a minimum clinically important difference of 15 points (pts.) was considered clinically significant. As another clinical PROM, the pain was assessed based on daily numerous rating scale (NRS) (0–10 pts.) recordings in the patient diary. We calculated PRE values from the mean of the first 14 days after baseline and POST values from the mean of the last 14 days of the intervention phase.

### Secondary Outcomes

Secondary outcomes were defined as functional parameters (i.e., vertical jump height) and tendon vascularity.

#### Functional Properties

Functional properties were assessed by estimating drop jump (DJ) and countermovement jump (CMJ) height as described previously [41]. Briefly, after a warm-up with up to 12 jumps of low to moderate intensity, five maximum effort CMJs and five DJs were performed (bare feet, hands akimbo, 1-min rest between repetitions). DJs were performed from a 15 cm box. Ground reaction forces were measured with two separate force plates at a rate of 1000 Hz (Kistler, Type 9260AA, 600 × 500 × 50 mm, Switzerland) linked to an analog digital converter (DAQ-System, USB 2.0, Type 5691A1). Data were recorded (BioWare Software, Type 2812A) and jump height was calculated based on the impulse–momentum method [62] for the CMJ and the flight-time method [63] for the DJ, using a custom-written MATLAB interface (version R2012a; MathWorks, Natick, MA, USA). For further CMJ and DJ analysis, the mean of the highest three jumps out of five attempts was used.

#### Vascularity

**Fig. 3:**
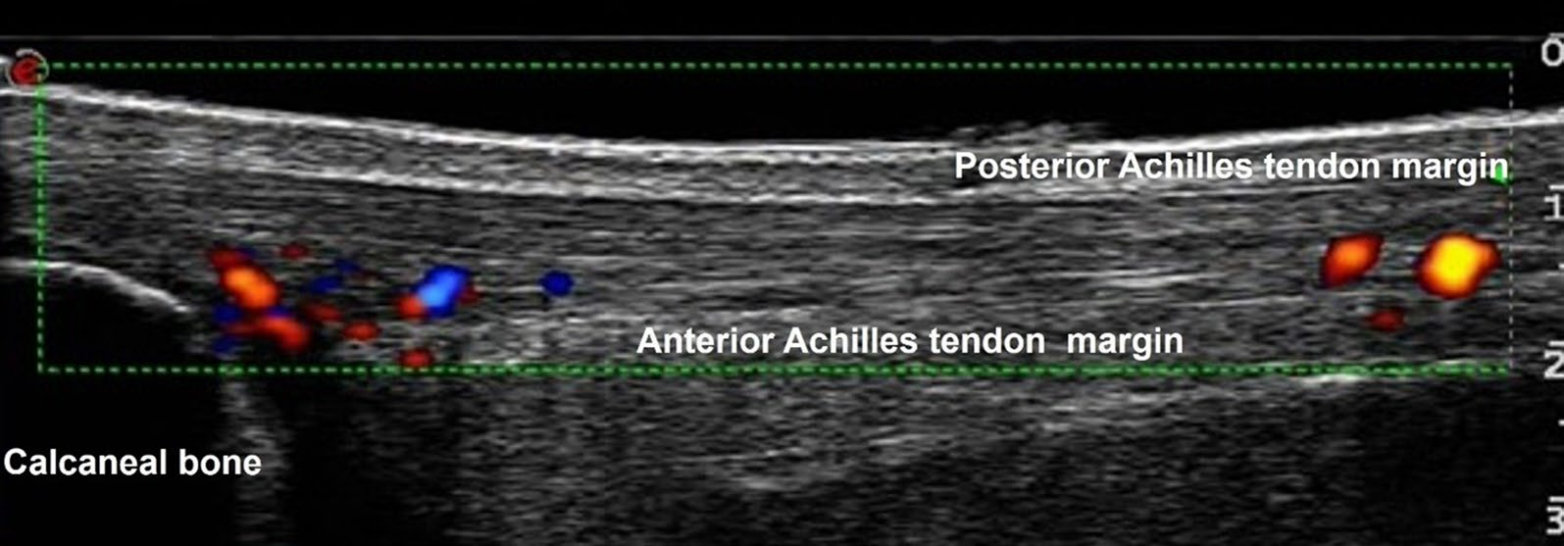
Sagittal power Doppler ultrasound scan of the intratendinous vascularity of a tendinopathic Achilles tendon. The green box (indicated by a thin dotted green line) shows the standardized rectangle frame (10.0 × 2.0 cm) of the region of interest in which the Doppler signal was visualized. Within this box, we analyzed vascularity within tendinous tissue only between the anterior margin (i.e., anterior margin of the Achilles tendon and the posterior margin of the calcaneal bone) and posterior margin of the Achilles tendon

Intratendinous vascularity was assessed in the sagittal plane with pulsed-wave power Doppler ultrasonography (7.1 MHz, My Lab60, Esaote, Genova, Italy) using the following settings: Wall filter 1, Density 1, Persistence 3, Pulse repetition frequency 750 Hz [36, 64]. Power and color gain were manually adjusted just below random noise level per participant [65] and recorded at PRE measurement to reapply for POST measurement. The transducer was aligned parallel to the tendon and applied with no pressure. The power Doppler transducer was positioned so that it visualized the proximal portion of the calcaneal bone and the Achilles tendon (Fig. 3). During measurement, participants were in the prone position with the knees extended and the ankle joint passively stabilized at 90° (i.e., tibia perpendicular to the foot) with relaxed plantar flexor muscles. Prior to the measurement, participants were advised to refrain from any intense exercise activity for 2 h [66]. For optimized visualization of tendon vascularity, three sets of 15 unilateral single-leg heel raises were performed before the measurement [67]. Three scans with a duration of 4 s each were recorded. The frame with both the highest signal activity and without any artifacts [68] was chosen for analysis. Analysis was performed using a custom-written MATLAB script (The MathWorks, version 2012, USA) quantifying the number of intratendinous colored pixels and converting them to mm^2^ (Fig. 3).

### Statistical Analysis

We examined the normality of data distribution with the Kolmogorov–Smirnov test. For all baseline between-group comparisons, we performed a one-way analysis of variance (ANOVA) (factor: group). For all PRE to POST comparisons except tendon CSA, a repeated measures ANOVA was conducted (within-subject factor: time; between-subject factor: group). For the PRE to POST comparisons of tendon CSA, we used a two-factor repeated measure ANOVA (factor 1: time; factor 2: localization, considering the 10% steps of the full free Achilles tendon length; between-subject factor: group. In case of a significant effect of interaction, a Bonferroni post hoc analysis was conducted, and adjusted p values were reported. The effect size concerning the effect of training was based on partial eta squared and calculated by Cohen’s f [69] and defined as follows: values of *f* = 0.10, *f* = 0.25, and *f* = 0.40 represent small, medium, and large effect sizes, respectively. The relationship of the dominant to the injured side was established with an adjusted Pearson’s contingency coefficient. For all statistics, the significance level was set at *α* = 0.05 and the software SPSS Statistics (IBM, version 21, USA) was used.

## Results

### Primary Outcomes

#### Mechanical and Material Properties

**Fig. 4:**
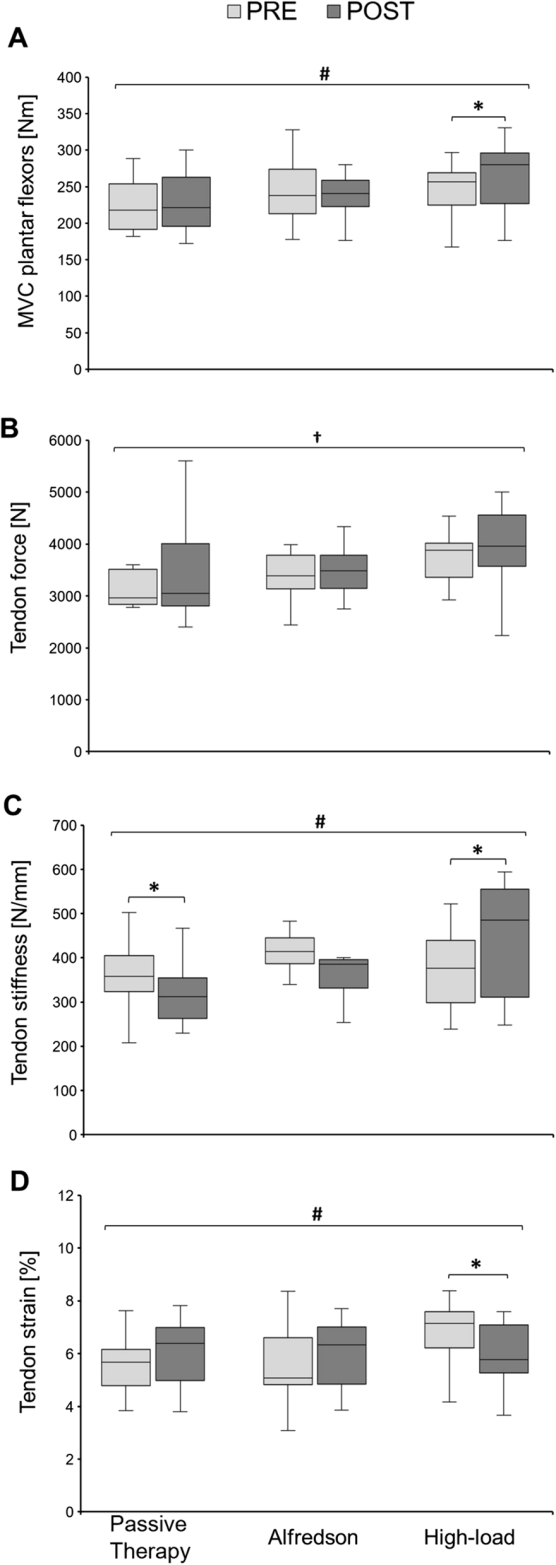
**A–D** Muscle strength and tendon mechanical properties of the symptomatic leg at baseline (PRE) and after the 12-week intervention phase (POST) for all three intervention groups. **A** Isometric maximum voluntary contraction (MVC) of the plantar flexor muscles. **B** Tendon force of the Achilles tendon. **C** Tendon stiffness of the Achilles tendon. **D** Maximum Achilles tendon strain. The horizontal line in the middle of the box is the median value of the scores, and the lower and upper boundaries indicate the 25th and 75th percentiles, respectively (median included). The largest and smallest observed values that are not outliers are shown by the lines drawn from the ends of the box to those values (whiskers). * indicates significant post hoc difference when compared to PRE (*p* < 0.05); † indicates significantly difference to PRE (*p* < 0.05) as a main effect of time; ^#^ significance with *p* = 0.05 as a group-by-time interaction effect

At baseline, maximum plantar flexor strength (i.e., MVC) (*p* = 0.335), tendon force (*p* = 0.698), tendon stiffness (*p* = 0.610) and maximum tendon strain (*p* = 0.146), stress (*p* = 0.331), Young’s modulus (*p* = 0.774) and tendon rest length (*p* = 0.134) did not significantly differ between groups (Fig. 4 and Table 3).

From PRE to POST, there was no significant time effect (*p* = 0.064), but a significant effect of time-by-group interaction for the MVC measurements of the plantar flexor muscles (*p* = 0.042; *f* = 0.41). Only in the High-load group, MVC increased by 7.2 ± 9.9% (*p* = 0.045) (Fig. 4A). There were no changes in MVC from PRE to POST in the Alfredson (*p* = 1.866) and the passive therapy group (*p* = 0.975).

Tendon force data demonstrated a significant main effect of time from PRE to POST (*p* = 0.008; *f* = 0.43) and no significant time-by-group interaction effect (*p* = 0.150), showing an overall mean increase of 6.79 ± 17.28% (Fig. 4B).

For tendon stiffness, there was no significant time effect (*p* = 0.887), but a significant time-by-group interaction effect (*p* = 0.0003; *f* = 0.70). Post hoc analysis revealed a significant increase in tendon stiffness of 20.1 ± 20.5% for the High-load group (*p* = 0.049) and a significant decrease in stiffness with -7.7 ± 21.2% for the passive therapy group (*p* = 0.042) (Fig. 4C). There was no change in stiffness for the Alfredson group (*p* = 0.306).

For maximum tendon strain, there was no significant time effect (*p* = 0.878), but a significant time-by-group interaction effect (*p* = 0.015; *f* = 0.48). The High-load group showed a significant maximum tendon strain decrease of -12.4 ± 10.3% (*p* = 0.001) (Fig. 4D), whereas the Alfredson group (*p* = 0.738) and the passive therapy group (*p* = 0.846) did not change.

For stress, there was no significant time effect (*p* = 0.52) or time-by-group interaction effect (*p* = 0.79).

Young’s modulus showed no significant main effect of time (*p* = 0.363), but a significant effect of interaction (*p* = 0.030; *f* = 0.45). The post hoc comparisons, however, did not show any significant changes from PRE to POST for the passive therapy (*p* = 0.159), the Alfredson (*p* = 0.327) and the High-load (*p* = 0.597) group (Table 3).

Tendon rest length showed no significant main effect of time (*p* = 0.784) and no time-by-group interaction effect (*p* = 0.734) (Table 3).

#### Morphological Properties

**Fig. 5:**
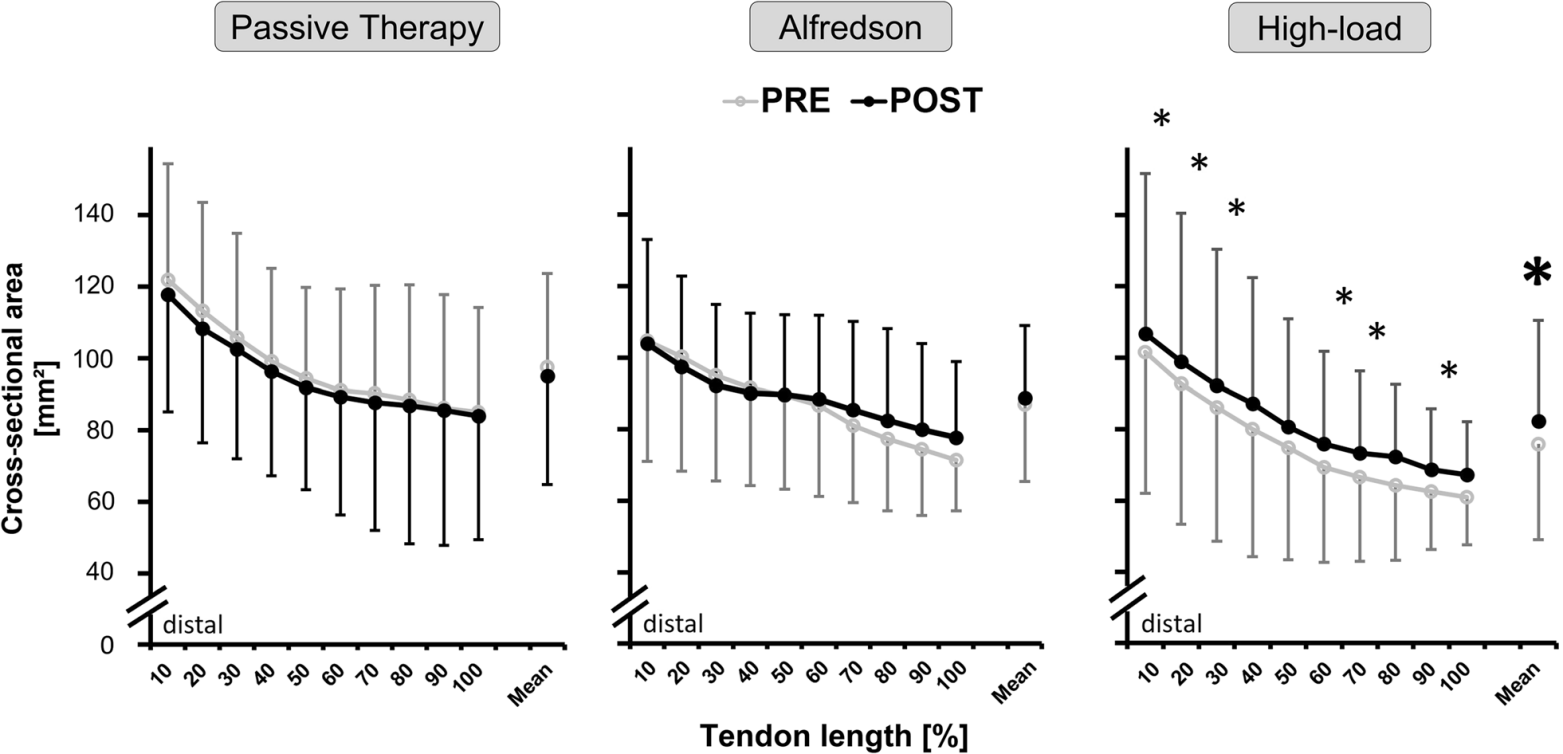
Cross-sectional area (CSA) values (mm^2^) of the symptomatic Achilles tendon measured by magnetic resonance imaging (MRI) at baseline (PRE) and after the 12-week intervention phase (POST) for each intervention group (Passive therapy *n* = 13, Alfredson *n* = 15, High-load *n* = 14). Data are presented as CSA values measured in 10% increments tendon length from distal to proximal alongside the free Achilles tendon and the total free Achilles tendon CSA mean ± standard deviation. * indicates significant post hoc difference when compared to the corresponding tendon region in the passive therapy and Alfredson group (*p* < 0.05). * indicates significant post hoc difference when compared to PRE (*p* < 0.05)

Mean Achilles tendon CSA did not significantly differ at baseline between the three groups (*p* = 0.253). From PRE to POST, there was a significant time effect (*p* = 0.021; *f* = 0.38) and a significant time-by-group interaction effect (*p* = 0.002; *f* = 0.62) for the mean Achilles tendon CSA. Post hoc analysis showed tendon hypertrophy across the whole tendon length (i.e., mean tendon CSA) for the high-load group from PRE to POST (8.98 ± 5.8%, *p* < 0.001). There were no mean tendon CSA changes from PRE to POST for the passive therapy (*p* = 0.681) and Alfredson (*p* = 0.765) group (Fig. 5).

#### VISA-A Score

**Fig. 6:**
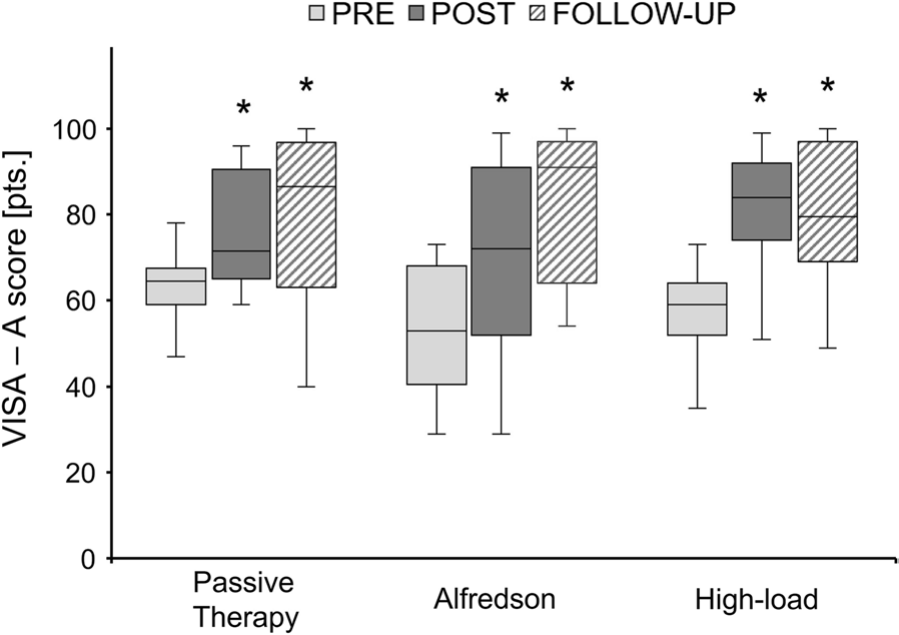
Victorian Institute of Sport Assessment (VISA)—Achilles (A) score at baseline (PRE), after the 12-week intervention period (POST) (Passive therapy *n* = 14; Alfredson *n* = 15; High-load *n* = 15) and 6 months after POST (FOLLOW-UP) (Passive therapy *n* = 14; Alfredson *n* = 13; High-load *n* = 13) for the three intervention groups measured in points (pts.) from 0 to 100. The horizontal line in the middle of the box is the median value of the scores, and the lower and upper boundaries indicate the 25th and 75th percentiles, respectively (median included). The largest and smallest observed values that are not outliers are shown by the lines drawn from the ends of the box to those values (whiskers). * indicates significant difference (*p* < 0.05) compared to PRE as a significant main effect of time

At baseline, the VISA-A score did not significantly differ between groups (*p* = 0.496) (Table 1). From PRE to POST, there was a significant main effect of time for the VISA-A score (*p* < 0.001; *f* = 1.34) with an overall clinically significant mean increase of 19.8 ± 15.3 pts. (Passive therapy group: 16.9 ± 14.7 pts., Alfredson group: 17.9 ± 16.4 pts., High-load group: 24.4 ± 15.0 pts.) and no significant time-by-group interaction effect (*p* = 0.375). From PRE to FOLLOW-UP, there was a significant main effect of time for the VISA-A score (*p* < 0.001; *f* = 1.25) with an overall clinically significant mean increase of 22.7 ± 18.4 pts. and no significant time-by-group interaction effect (*p* = 0.869) (Fig. 6). From POST to FOLLOW-UP, the VISA-A score did not significantly change (main effect of time: *p* = 0.168; time-by-group interaction effect: *p* = 0.412).

#### Pain

At baseline, average pain did not significantly differ between groups (*p* = 0.957). From PRE to POST, there was a significant main effect of time (*p* < 0.001; *f* = 0.66) and no significant interaction effect (*p* = 0.166) demonstrating a significant reduction in pain for all three groups by −0.55 ± 0.9 NRS pts. (Table 2).

**Table 2:** Pain and weekly exercise activity level at baseline (PRE) and after completion of the 12-week intervention phase (POST) for the three intervention groups based on the training diary

Parameter	Passive therapy		Alfredson		High-load	
	PRE Mean ± SD	POST Mean ± SD	PRE Mean ± SD	POST Mean ± SD	PRE Mean ± SD	POST Mean ± SD
Pain NRS [0–10]^A^	2.6 ± 1	1.6 ± 1.1*	2.4 ± 1.6	2.1 ± 1.7*	2.5 ± 1.8	2 ± 1.7*
Activity level [h]^A^	2.98 ± 2.92	1.67 ± 1.66	3.77 ± 4.04	3.89 ± 4.05	3.80 ± 3.79	3.31 ± 2.97

Presented as mean ± standard deviation (SD)

NRS, numeric rating scale; h, hours

*Significant difference (*p* < 0.05) compared to PRE as a main effect of time

^A^Average of the first two weeks (PRE) and the last 2 weeks (POST) of the intervention period

### Secondary Outcomes

#### Jump Performance

At baseline, there were no significant differences between groups for the CMJ (*p* = 0.762) and DJ (*p* = 0.659). From PRE to POST, there was a significant time effect (*p* = 0.005; *f* = 0.47) and no time-by-group interaction effect (*p* = 0.219) for CMJ height showing an overall decrease of −0.63 ± 4.07 cm. From PRE to POST, there was no significant time effect (*p* = 0.319) and no time-by-group interaction effect (*p* = 0.820) for DJ height (Table 3).

**Table 3:** Function, material properties, intratendinous vascularity and rest length measured at baseline (PRE) and after completion of the 12-week intervention phase (POST) for the three intervention groups presented as mean ± standard deviation (SD)

Parameter	Passive therapy		Alfredson		High-load	
	PRE Mean ± SD	POST Mean ± SD	PRE Mean ± SD	POST Mean ± SD	PRE Mean ± SD	POST Mean ± SD
CMJ height [cm]	26.09 ± 5.1	25.08 ± 5.6*	26.73 ± 4.5	26.53 ± 4.9*	27.39 ± 4.5	25.9 ± 4.0*
DJ height [cm]	21.66 ± 4.9	20.51 ± 5.1	23.75 ± 5.9	23.4 ± 5.7	22.34 ± 7.4	22.21 ± 5.5
Stress [MPa]	39.6 ± 17.3	40.9 ± 16.9	41.1 ± 12.2	40.8 ± 13.1	48.0 ± 16.6	48.9 ± 15.2
Young’s modulus [GPa]	0.90 ± 0.40	0.79 ± 0.29^#^	0.92 ± 0.26	0.84 ± 0.33^#^	0.99 ± 0.31	1.10 ± 0.40^#^
Vascularity [mm^2^]	9.09 ± 18.10	5.96 ± 11.70	4.03 ± 5.20	5.90 ± 10.90	2.08 ± 3.90	6.02 ± 11.30
Rest length [mm]	203.8 ± 26.2	202.3 ± 21.5	187.9 ± 15.1	190.0 ± 17.5	199.9 ± 22.9	200.8 ± 24.2

Countermovement jump (CMJ) height and drop jump (DJ) height data are presented as a mean of three highest out of five attempts. Stress, Young’s modulus, vascularity and rest length data are presented for the injured leg

*Significantly different to PRE with *p* < 0.05 as a main effect of time

^#^Significance with *p* < 0.05 as a group-by-time interaction effect

#### Vascularity

Intratendinous vascularity of the injured Achilles tendon did not differ between groups at baseline (*p* = 0.216). From PRE to POST, there was no significant main effect of time (*p* = 0.549) and time-by-group interaction effect (*p* = 0.151) (Table 3).

### Training Diary Analysis

#### Compliance

Compliance was 82.7 ± 29.5%, 80.8 ± 17.4% and 90.1 ± 15% in the passive therapy, Alfredson and High-load group with no significant difference between groups (*p* = 0.458). The compliance for additional passive therapy appointments in the exercising groups was 30 ± 39.6% and 35 ± 32% for the Alfredson and the High-load group. No impeding incidents caused by the interventions were reported.

#### Activity Level

Weekly activity levels, averaged over the first two weeks of the intervention period, did not significantly differ between groups (*p* = 0.800). Comparing the first to the last two weeks of the intervention period, there were no significant main effects for time (*p* = 0.112) and no group-by-time interaction (*p* = 0.318) (Table 2).

#### Progression

From PRE to POST, the Alfredson group increased training load by 12.0 ± 15.1 kg (based on the training diary) ranging from 0 to 55 kg and the High-load group increased load by 10.0 ± 13.2 kg with a range of −9 to 38 kg. There was no significant difference in load progression between groups (*p* = 0.728).

#### Passive Therapy Treatment

The physiotherapists applied a variety of non-lower-limb loading treatment practices, differing from patient to patient and including manual therapy/joint mobilization/tissue stretching (37%, 47% and 35%), core stability (8%, 0% and 8%), massage/deep friction/foam rolling (35%, 7%, and 26%), electro-/sono-/thermotherapy (20%, 46% and 24%) and unspecified (0%, 0% and 7%) for the passive therapy, Alfredson and High-load group, respectively.

### Laterality

The analysis of the strength of the correlation between the leg laterality of the dominant side and the injured side did not reveal any relationship (corrected Pearson's contingency coefficient *C*_korr_ = 0.066; *p* = 0.542).

## Discussion

As hypothesized, by increasing Achilles tendon stiffness and tendon CSA, the high-loading protocol yielded similar responses in male tendinopathic patients as previously observed in male healthy tendons. These responses in the High-load group were accompanied by significant clinical improvements. Nevertheless, as VISA-A scores increased not only in the High-load group, but in all groups irrespective of the intervention protocol and the therewith associated morphological and mechanical adaptations of the Achilles tendon, the effectiveness of an exercise treatment protocol in terms of clinical improvement was not exclusively linked to its capacity in inducing structural tendon adaption. Thus, our hypothesis that superior structural adaptations will lead to superior clinical properties cannot be confirmed. Although the high-loading protocol did not result in acute superior therapeutic effects concerning the superior improvement in clinical and functional parameters when compared to standard eccentric exercises or passive therapy, prolonged benefits may be superior, as the mechanical and morphological adaptations in the High-load group may improve the tissue integrity and protect the tendon from further strain injury risk. As mechanical and morphological Achilles tendon properties remained unchanged in the Alfredson’s group and tendon stiffness even decreased in the passive therapy group, the aforementioned protection from strain injury might be reduced.

Chronic Achilles tendinopathy and the therewith associated pathological changes in tendon structure or metabolism do not seem to impair the Achilles tendons’ adaptive capacity to respond to intense mechanical loading. Indeed, the increase in Achilles tendon CSA of 9% observed after 12 weeks of high-loading in our tendinopathic patients was not smaller than the mean Achilles tendon CSA hypertrophy of 4.2% observed in healthy adults after 14 weeks of training [28]. Comparable region-specific increases in asymptomatic tendon CSA were observed in the patellar tendon [70, 71] and Achilles tendon [26] after high-loading exercise. The increase in Achilles tendon CSA has been detected nearly across the whole length of the tendinopathic tendon as it did in the healthy participants in previous studies [28], indicating that CSA hypertrophy is most likely caused by an evenly distributed accumulation of collagenous material and not by localized swelling or edema. Moreover, the increased Achilles tendon stiffness after the high-load intervention also supports the argument for an anabolic tendon response and against swelling or edema. While several studies have reported morphological adaptations after loading exercise in healthy populations [26–28, 70, 71], so far there has been no evidence for therapeutic exercise interventions inducing tendon hypertrophy in Achilles tendinopathy as recently pointed out in a review [72].

This high-loading-induced Achilles tendon hypertrophy is likely to have caused the co-occurring increase in tendon stiffness of 20%. Again, in healthy tendons similar adaptations in tendon stiffness have been observed. In healthy Achilles tendon, stiffness increased by 36% [26], 17.1% [27] and 57% [28] after 14 weeks of high-loading.

As the tendon cannot be trained in isolation, the high-loading protocol also leads to adaptations in muscle strength. Plantar flexor MVC increased by 7.2% from PRE to POST in the High-load group, which corresponds to gains to the same high-loading protocol in healthy populations who trained at home with the mobile device (10%, [31]) or under laboratory conditions on a dynamometer (7%, [46]). The aforementioned structural adaptations of the Achilles tendon have not been affected or biased by a possible correlation between the leg laterality of the injured leg and the leg laterality of the dominant leg.

Overall, the magnitude of changes in the triceps surae muscle–tendon unit of Achilles tendinopathy patients in response to 3 months of home-based high-loading tendon training is comparable to those observed in healthy participants. Thus, the exercise response of healthy Achilles tendon tissue seems to be transferable to tendinopathic tissue. In sum, despite pathological tissue changes, the Achilles tendons of tendinopathy patients benefit in the same way as healthy Achilles tendons from high-loading tendon training.

The significant structural and clinical benefits of high-load tendon training are apparent short term and may prolong. The increased training-induced tendon stiffness will decrease strain at a given load and may thus contribute to preventing strain-induced (micro)damage and tendon injuries. This protective effect is indicated by the 12% decrease in maximum strain in the High-load group post-intervention. Indeed, the effect of a specific tendon training on maintaining tendon tissue integrity and preventing tendon pain has recently been demonstrated for the patellar tendon, highlighting the potential of high-loading tendon training as preventive measure: Two high-loading tendon training sessions per week over the course of one year reduced the prevalence of tendon pain in adolescent handball players compared to a control group, who continued with their usual training [22]; three sessions per week over one year maintained tissue integrity in adolescent basketball players as determined by spatial frequency analysis, while impairments of tendon micromorphological integrity with higher strain were evident in the control group [23]. As exclusively in the tendon-trained group tendon stiffness increased and high levels of strain decreased [23], it is likely that it is indeed the increased stiffness that protects the tendon from strain-induced microdamage and pain. It has to be considered though that there may be differences in the adaptational response of an adolescent compared to an adult tendon. However, if we assume, that those findings are transferable to the Achilles tendon of adults, it might be conceivable that the increased stiffness and the reduced strain resulting from the high-load intervention in our tendinopathy patients may have prolonged effects on tendon health.

The high-load exercise protocol significantly improved pain and function in Achilles tendinopathic patients without demonstrating a superior clinical outcome when compared to passive therapy (i.e., the passive therapy group) or Alfredson’s protocol. Thus, superior structural adaptations such as increased Achilles tendon stiffness and reduced tendon strain did neither directly post-intervention nor 6 months later at follow-up appear to directly translate in a superior reduction in tendinopathy symptoms or an improvement in function (jump performance). Indeed, several studies have shown, that it is difficult to link structural changes to pain or clinical outcome, with a review concluding that structural and clinical findings in tendinopathy do not necessarily relate to each other [73]. In addition, a recent review of exercise-induced tendon adaptation reported no correlation between change in tendon thickness and clinical outcome (i.e., self-reported pain and function), highlighting a lack of evidence concerning the effect of improved mechanical properties on clinical outcome particularly in Achilles tendinopathy [72].

In our trial, we found that in all groups a significant reduction in pain (NRS) went parallel with a significant improvement in the VISA-A score without differences between groups. The applied passive therapy, which was available for all groups due to ethical reasons, consisted of > 90% of manual therapy treatment which is known to have hypoalgesic effects [74–78]. It may thus have affected both NRS and VISA-A outcomes in all groups. Moreover, on-site support by a physiotherapist might add to pain reduction and therefore improvements in VISA-A score due to psychological factors [79]. The much higher adherence of the passive therapy group (82.7%) compared to Alfredson and High-load group (30.0% and 35.0%) in addition to a much lesser activity level of the passive therapy group (although not significantly different) (Table 2) might as well contribute to the POST NRS and VISA-A score levels in the passive therapy group.

Furthermore, conflicting results about intratendinous neovascularization which is supposed to coincide with tendinopathy. On the one hand, a correlation of neovascularization with clinical severity has been reported [80] and there is evidence that a reduction in neovessels by treatment with sclerosing agents improves clinical findings [81]. On the other hand, no correlation between neovascularization and pain or function (i.e., VISA-A score) has been found at baseline in an intervention trial with 37% of the symptomatic tendons showing no neovascularization [82], demonstrating the difficulty when concluding from neovascularization to clinical severity [83].

Structural adaptation also does not directly translate into an improvement in function or better performance, even though higher tendon stiffness is reported to contribute to improved jump performance [84]. While in healthy adults the home-based application of the same high-load protocol led to increased DJ height [41], in the tendinopathy patients of this study no change in DJ height was observed and the CMJ height even dropped post-intervention. Moreover, while Achilles tendinopathy is said to be accompanied by a decrease in functional capacity such as vertical jumping [85], a clinically effective exercise therapy that was associated with significant improvements in VISA-A score did similarly not result in improved CMJ performance [49]. These inconsistencies might indicate the complexity of translating isometric strength gain into functional movements where the stretch-shortening cycle is involved [86], while the discrepancy of translating structural adaptation into function might even be higher in tendinopathic tendons.

Every therapeutic intervention in our study was clinically effective and applicable. Nevertheless, the high-loading protocol offers some advantages over passive therapy and standard eccentric exercises.

Although passive therapy was equally effective in improving the VISA-A score, tendon stiffness decreased significantly post-intervention in this group, indicating tissue deterioration and potentially increasing the risk for future strain-induced tendon injuries. This decrease in tendon stiffness could be related to reduced mechanical stimulation of the tendon, as the passive therapy intervention did not apply high strains to the tendon.

In contrast to passive therapy, the mechanical stimulation provided by the eccentric exercise intervention appeared to be sufficient to maintain tendon stiffness. Despite a more than fifteen times longer loading time per week when compared to the high-loading protocol (Table 1), the eccentric exercises did, however, not result in structural adaptations, suggesting that the magnitude of the stimulus may not have reached the threshold to generate hypertrophic responses. This is in line with the literature reporting no effect on mechanical properties (i.e., increase in tendon stiffness) after eccentric exercise in healthy tendons [34, 35, 87].

Although eccentric exercises are still the gold standard due to the strong evidence base [88], specific benefits of the eccentric exercise as a superior tendon strengthening protocol may be questioned as peak tendon forces do not differ between concentric (i.e., toe raising) and eccentric (i.e., toe lowering) contractions [89]. In terms of structural adaptations and the therewith associated potential strain-induced injury preventive effects, the high-loading protocol appears to be superior compared to both passive therapy and eccentric exercises.

### Limitations

We investigated a male patient population as gender might be a confounding factor [49] leading to gender-specific effects post-intervention [90] conceivably due to differences in Achilles tendon stiffness and rate of tendon hypertrophy [91]. These differences might have led to potential between-group heterogeneity. Thus, the effectiveness of the high-loading protocol needs to be verified with a female population and potentially adapted, to be applicable to females. In addition, as tendinopathy patients are generally a very heterogeneous group (e.g., highly differing in activity status and BMI), the high-loading protocol may not necessarily lead to similar improvements for the entire spectrum of Achilles tendinopathy patients when compared to our study sample.

We assessed both insertional and mid-portion tendinopathy without differentiation. This way, our results might inform clinicians dealing with chronic Achilles tendon disorder and support their decisions for either location. As the locations were equally distributed (Table 1), we did not expect any major outcome inconsistencies and thus, we do not think that the validity of our results is affected by the ratio of insertional and mid-portion tendinopathy in the different treatment groups. Nevertheless, we acknowledge that due to differences in structure [92], particular rehabilitation approaches for either location were recommended [48] as outcomes may differ [93].

We assume that improved structural adaptations do have the potential for prolonged benefits as training-induced overload injury might be reduced. As our study design was not able to adequately assess long-term effects, future research with a more comprehensive (i.e., return to sport) follow-up in addition to a much longer monitoring time period should further establish this empirically.

The allocation-to-group was not predictable. As one of the three assessors knew the allocation sequence (G.R.), entirely concealed allocation could not be guaranteed. Therefore, a methodological risk of bias (i.e., internal validity) cannot be fully excluded. However, there were no significant differences between groups at baseline in none of the parameters which might give statistical support that the allocation method did not establish allocation bias. In addition, ensuring a strict standardized assessment procedure without disclosure of our study hypothesis at any time might have reduced potential bias. Blinding (to groups) of patients and therapists was precluded, as patients must know about their allocation as well as their supervising therapists to conduct and support treatment properly which is a common methodological approach [94].

A physiotherapy certificate as well as a medical referral/receipt was mandatory to apply this intervention whereas for the application of the Alfredson and the High-load intervention it was not as it was home-based. However, it is self-evident that Alfredson and High-load interventions as exercise modalities are certainly within the scope of physiotherapy treatment, and based on our results, we encourage the application of high-loading by physiotherapists.

Passive therapy treatment might impact clinical outcomes. However, a control group without any therapeutic modalities at all is considered to be unethical [95]. The therapeutic modalities of our control (i.e., passive therapy) group thus aimed to control one of our primary outcomes (i.e., structural tendon adaptation). As the lack of mechanical loading of the Achilles tendon presumably would not lead to any structural adaptations, our passive therapy group controlled the two active exercise groups (i.e., High-load and Alfredson). Moreover, we aimed to control passive therapy beforehand by thorough instructions (i.e., letter to the physio and medical referral) and afterward due to the training diary reported by each patient. Thus, we considered the therapy applied within the utilized appointments in all three groups to be mostly (i.e., > 90%) of passive nature and to not have positive effects on structural tendon adaptation.

Clinical assessment was performed by recording the VISA-A score and a daily average for pain (NRS). Although the VISA-A score is the most widely used assessment in Achilles tendinopathy, the current literature alluded to some weaknesses of the VISA-A score questioning its validity and adequacy [96]. Moreover, recent literature recommends the assessment of nine core domains reflecting physical, psychosocial and overall status/life impact factors to better estimate treatment effects [97].

## Conclusions

The application of the high-load protocol, which has already been shown to be reliable and effective in healthy participants [41], showed very promising results as an exercise therapy option in male Achilles tendinopathy patients. High compliance and no reported impeding incidents suggest that this therapeutic approach is highly feasible. Structural adaptations, potentially leading to prolonged health benefits, are in favor of this therapeutic approach. In addition, its home-based application and its time-saving implementation, compared to the eccentric exercise protocol (Table 1), may be beneficial for high adherence. Thus, we recommend the application of high-loading in Achilles tendinopathy patients as an effective (alternative) therapeutic protocol for clinicians and therapists in Achilles tendinopathy rehabilitation management.

## Data Availability

The datasets generated during and/or analyzed during the current study are available from the corresponding author upon reasonable request.
